# Cultures of Victory and the Political Consequences of Foundational Legitimacy in Croatia and Kosovo

**DOI:** 10.1177/0022009419838045

**Published:** 2019-10

**Authors:** Mieczysław P. Boduszyński, Vjeran Pavlaković

**Affiliations:** Pomona College, USA; University of Rijeka, Croatia

**Keywords:** Croatia, Former Yugoslavia, Kosovo, politics of memory, statebuilding, war veterans

## Abstract

What are the consequences of a culture of victory in countries undergoing new state formation and democratic transition? In this article, we examine ‘foundational legitimacy,’ or a hegemonic narrative about the way in which a new state was created, and the role particular groups played in its creation. We argue that the way in which victory is institutionalized can pose a grave threat to the democratic project. If reconciliation and democratization depend of integrating losers into the new order and recognizing plural narratives of state formation, then exclusivist narratives based on foundational legitimacy pose a direct challenge to both. We focus on two Yugoslav successor states, Kosovo and Croatia. For both cases, we trace how appeals to ‘foundational legitimacy’ by groups that claim a leading role in the struggle for independence fostered a politics of exclusion, which ran counter to both the spirit of democracy. In Croatia, foundational legitimacy was partly challenged after 2000 by reformist political forces, though more recently it has re-appeared in political life. In Kosovo, foundational legitimacy was never successfully challenged and continues to shape political dynamics to the present day.

How do foundational cultures of victory become routinized in the politics and institutions of new states? What effect does ‘foundational legitimacy,’ a hegemonic narrative about the way in which a new state was created and the role particular groups played in its creation, have on the prospects for democratic transition? How are foundational narratives challenged and adapted over time? Here we address these questions in the context of two newly-independent states that emerged from the former Yugoslavia, Croatia and Kosovo. In both of these states, movements born of wars of independence came to dominate the post-Yugoslav transition, and claimed exclusive ownership over the foundational narrative. In both Croatia and Kosovo these movements became embedded in institutions – political parties and veterans’ organizations in particular – and used their respective claims of ownership over foundational legitimacy to seize power and resources. Owing to the existence of dominant foundational narratives, Croats and Kosovar Albanians as both victims and victors in their respective wars of independence have been often unwilling to come to terms with their third role, as perpetrators of abuses.

As the editor of this special issue suggests, ‘cultures of victory’ can be divisive and unsettling forces in any postwar society. But they are particularly unsettling in the context of the formation of new states such as Kosovo and Croatia. This is because the nature of ‘victory’ in a war of secession leading to independence is destined to play a central role in the creation of narratives about a new state’s birth. Such victory narratives become, in turn, key parts of foundational myths, enumerated in school history textbooks, celebrated on holidays, and engraved on memorials.

In new states, there exists an acute need to locate tangible and meaningful content (a ‘usable past’)^1^ for the national narrative so as to build *state and national identity.* But the manner in which a new state is conceived is also a delicate subject, not only for the losing side in an independence struggle, but even within the winning coalition.

There are major incentives for groups who played a leading role in the conflict to lay claim to foundational legitimacy as ‘memory entrepreneurs’^2^, for it can be easily converted to political and economic power, as well as social prestige. For these same reasons, the groups that played this leading role might use their claims to foundational legitimacy as a weapon with which to fight competitors in the struggle for political and economic influence. They can portray any criticisms directed against them as an affront to the liberation struggle itself, a high bar to overcome and one that can easily be used to silence dissent. In the worst-case scenario for the development of liberal democracy, claims to foundational legitimacy can be used to marginalize and exclude competitors from meaningful participation in political life. Moreover, claims to foundational legitimacy can be used to challenge constitutional and electoral legitimacy. Finally, groups claiming foundational legitimacy have an incentive to fiercely oppose any effort to defy the content of the liberation narrative. Foundational legitimacy rests on the ‘purity’ of this narrative, which must be cleansed of any crimes committed by those who fought for independence. In sum, challenges to the foundational legitimacy espoused by groups claiming a leading role in the independence struggle are seen as a threat to the prestige, privilege, and political and economic power these groups enjoy.

These dynamics, we will show, have been a feature of political life in both Croatia and Kosovo since independence (in Croatia, independence in 1991; in Kosovo, de facto self-rule after the 1999 NATO intervention and a declaration of full independence in 2008), where individuals and groups instrumentalized claims to foundational legitimacy so as to solidify their political positions, extract resources from the state, cover up corruption, and avoid meaningful steps toward reconciliation, all of which had an adverse effect on democratization.^3^

Foundational legitimacy, as suggested above, tends to be intricately linked to particular groups or factions and their claims of heroic exploits during a war of independence. In the case of Croatia, we analyze groups such as the Croatian Democratic Union (*Hrvatska Demokratska Zajednica,* HDZ), the Croatian Army, and veterans’ groups, all of which have used the foundational myth of the ‘Homeland War’ as a source of privilege and political clout. In the case of Kosovo, we analyze the Kosovo Liberation Army (UÇK, in Albanian, *Ushtria Çlirimtare e Kosovës,*) and its principal role in fighting a guerrilla war against Serbian domination. Two political parties led by former militia commanders emerged from the UÇK, the Democratic Party of Kosovo (*Partia Demokratike e Kosovës,* PDK) and the Alliance for the Future of Kosovo (*Aleanca për Ardhmërinë e Kosovës*, AAK). In both Croatia and Kosovo, narratives expounded by these dominant groups departed from the actual conduct of liberation struggles, which included documented war crimes, but the foundational myths they helped create and promote sustained their hegemony in politics and institutions for many years.

In what follows, we trace the use of foundational legitimacy as deployed by the HDZ and UÇK/PDK, as well as veterans’ organizations in both countries. We note its political consequences, showing how it becomes embedded in parties and institutions that in turn use their claims to ownership over the victory memory to bid for scarce resources, capture state institutions, and cover up corruption and criminality. We also show how claims to foundational legitimacy are used to exclude political opponents, minorities, and others, harming the process of democratization. Thus, we aim to document how a culture of victory can be a grave threat to the liberal democratic project in new and transitional states like Kosovo and Croatia.

Our analysis proceeds as follows. We begin with the case of Croatia, and describe how the Croatian War of Independence (1991–5), or ‘Homeland War’ (*Domovinski rat*), resulted in a new culture of victory that was quickly monopolized by President Franjo Tuđman’s HDZ. We show that although foundational legitimacy was to some extent successfully challenged in the early 2000s by a new leadership intent on moving the country toward membership in the European Union (EU), certain groups, especially a number of influential veterans’ organizations, continued to use foundational legitimacy as a way to claim ownership over the Homeland War and dominate memory politics more generally. We then analyze the case of Kosovo, where the UÇK guerrilla movement successfully converted its battlefield exploits in the war for independence into political capital. We trace how Kosovar groups and individuals espousing a culture of victory came to dominate the political and economic space throughout the 2000s and 2010s, and how their appeals to foundational legitimacy as cover for corruption and state capture lowers the quality of its democracy.

Any analysis of post-Yugoslav cultures of victory in Croatia must start in the person of Franjo Tuđman, a former Partisan officer and dissident historian, who effectively used nationalist discourse to mobilize electoral support in the first multiparty elections in Croatia in 1990. Tuđman’s combined communist credentials and Croatian nationalism made him appealing to a wide spectrum of voters when it became clear that the ruling establishment in Croatia was unwilling to resolutely resist Milošević’s power grab that threatened to turn Yugoslavia into centralized Serbian state. Tuđman’s political ideology of ‘national reconciliation’ adopted an ambivalent attitude towards the Second World War-era fascist Ustaša regime, combining formal denunciation with the re-inclusion of its supporters in national discourse.^4^ Even as he engaged in such revisionism, Tuđman was able to recruit elements of the security services, JNA officer corps, and Communist Party members in the battle for an independent Croatia that was based upon anti-communist and anti-Yugoslav rhetoric.^5^

The subsequent conflict in Croatia resulted in not only the establishment of an independent state, but a new culture of victory symbolically founded upon the sacrifices of Croatian soldiers (*branitelji*, or defenders),^6^ who were given numerous privileges in the postwar state. One side of the Homeland War narrative focused on victimization, while the other side incorporated the victory narrative based upon the successful military operations against Serbs universally labelled as aggressors and traitors.

The first year of the war, epitomized by the destruction and occupation of the town of Vukovar, cemented the victimization narrative as the well-equipped JNA supported rebel Croatian Serbs in taking territory. ‘As a symbol of the sacrifice of the Croatian people and of the birth of the modern Croatian state,’ explains sociologist Kruno Kardov, ‘Vukovar became an imagined place, disengaged from time and space and created at a great distance from its organic surroundings.’^7^ The ethnic cleansing campaign by Serb forces resulted in the creation of the unrecognized Republic of Serbian Krajina (RSK – *Republika Srpska Krajina*) on approximately 30 per cent of Croatian territory. After several years of smaller military actions and attempts to re-incorporate the RSK into Croatia through negotiations, the Croatian Army (HV – *Hrvatska vojska)* launched two massive offensives in 1995 (Operations Flash and Storm), which were able to liberate the majority of the occupied territories but also resulted in the exodus of 150,000–200,000 Serbs from Croatia.^8^ After the shift in the balance of power on the battlefield and the intervention of the international community, the Dayton Accords (1995) ended the conflicts in Croatia and Bosnia-Herzegovina, while the Erdut Agreement (1995) formulated the peaceful reintegration of the Eastern Slavonian region.

The Croatian victory in the Homeland War undoubtedly secured Tuđman’s place in history as the founder of the modern independent state, and his HDZ as the undisputed state-building party (*državotvorna stranka*), the owner of foundational legitimacy. Tuđman’s second inauguration as president in 1996 deliberately took place on 5 August, the first anniversary of the HV’s decisive capture of Knin, which has since been celebrated annually as Victory and Homeland Thanksgiving Day and the Day of Croatian Veterans. According to historian Ivo Goldstein, the HV ‘was a kind of party army for the HDZ,’ a party which regularly had active generals on its election lists during the 1990s.^9^ Issuing veteran status also became a key pillar of the HDZ’s creation of a vast patronage system that guaranteed votes, ensured a loyal army of supporters willing to take to the streets, and allowed access to resources, while simultaneously dedicating themselves to the dominant historical narrative. Under Tuđman, Croatia was routinely criticized by international organizations for authoritarianism, corruption, violation of human rights, and refusal to cooperate with the International Criminal Tribunal for the former Yugoslavia (ICTY), established in 1993.^10^ The war years and immediate postwar period also resulted in the dramatic transformation of the socialist economy into a crony capitalist one that benefited those close to the ruling HDZ.^11^

Under Tuđman, who dominated Croatian politics until his death in 1999, the culture of victory was not only omnipresent, but justified the collective guilt applied to all Croatian Serbs as well as those in the political opposition, civil society, or international community who dared to criticize Tuđman or the Croatian Army, which had not waged such a pure and unblemished war as portrayed in the state-controlled media. A few brave journalists and human rights activists continued to draw attention to the disappearance of Serb civilians in places such as Gospić, Osijek, Sisak, and even Zagreb, as well as the systematic destruction of Serb property following the military operations of Medak Pocket (1993), Flash, and Storm. Even when pressured with international isolation and sanctions, the Tuđman administration refused to adequately investigate crimes committed by Croatian forces, creating a sense of impunity for those with *branitelj* status. On 19 April 1996, under international pressure, the Croatian *Sabor* (parliament) enacted the Constitutional Law for Cooperation of the Republic of Croatia with the ICTY, recognizing the court’s jurisdiction over crimes committed in the former Yugoslavia since 1991.^12^ Tuđman reluctantly handed over several Bosnian Croats indicted for war crimes by the ICTY, but he steadfastly refused to allow independent investigations into suspected violations in Croatia.^13^ Although on paper Croatia the rule of law was functioning, in practice foundational legitimacy trumped domestic and international laws and treaties regarding human rights, freedom of speech, and an independent judiciary.

The death of Tuđman and the election of a coalition of six opposition parties (*šestorka*), led by Prime Minister Ivica Račan of the Social Democratic Party (SDP), and Stjepan Mesić as president in early 2000 represented a sea change in Croatia regarding the internal organization of the fledging state, relations with the international community, and willingness to tackle difficult issues related to the Homeland War. On 14 April 2000, the new government issued a declaration reaffirming Croatia’s commitment to fulfill its obligations to the Tribunal, with one notable change from all previous declarations; Operations Flash and Storm were no longer declared to be under the exclusive jurisdiction of Croatia. Račan’s government recognized the right of the Tribunal to ‘begin proceedings determining the responsibility of war crimes committed during and immediately after the end of the Homeland War.’^14^ The willingness of the left-center government and president to fully prosecute those on the Croatian side suspected of war crimes and other abuses soon sparked a backlash by veterans’ organizations that would hang over the administration during its entire mandate. These organizations, and the right-wing political parties that supported them, resisted efforts to impose the rule of law over the foundational legitimacy that had dominated until the acquittal of Generals Ante Gotovina and Mladen Markač in 2012, at which point a new phase of veteran mobilization took place.^15^

In the period 2000–12, the protests by those groups who believed foundational legitimacy overruled the rule of law was directed at attempts to hand over Croatian officers to the tribunal in The Hague as well as to disrupt domestic trials of suspected Croat war criminals. The most common phrases that could be heard during these protests were that the leftist government and the international community wanted to ‘criminalize the Homeland War’ and that the arrest of Croatian Army members would damage the dignity of Croatia’s victory, and presumably raise questions about the legitimacy of the state itself. However, Croatia was obligated to investigate all cases of war crimes according to both its own constitution and numerous international treaties, so a more reasonable explanation was not that veterans feared Croatia would be compromised internationally, but rather that their privileges based on foundational legitimacy would potentially be threatened. The first serious threat from veteran groups and still-active members of the HV came in September 2000, when 12 generals wrote an open letter to President Mesić in which they condemned the criminalization of the Homeland War. Mesić responded quickly and resolutely, immediately retiring the seven active generals (Davorin Domazet Lošo, Mirko Norac, Krešimir Ćosić, Ante Gotovina, Damir Krstičević, Ivan Kapular, and Miljenko Filipović) and reasserting civilian control over the military.^16^ As a reaction to volatile public debates over how the country should proceed regarding potential war criminal prosecutions of HV members, Račan’s government attempted to appease the right wing by issuing the Declaration of the Homeland War, which the *Sabor* enacted on 13 October 2000. The declaration establishes that ‘the Republic of Croatia waged a just, legitimate, and defensive war of liberation, and not an aggressive or expansionist war against anybody, in which it defended its territory against Greater Serbian aggression within its internationally recognized borders.’^17^ Although the Declaration established an official narrative that complied with the pre-existing culture of victory developed under Tuđman, noticeably overlooking Croatian operations in Bosnia-Herzegovina, it did little to convince the most politicized veteran groups that Račan and Mesić would continue Tuđman’s policies based upon foundational legitimacy. Nevertheless, Mesić’s dismissal of the generals was a critical juncture in the post-independence history of Croatia, when the institutionalized dominance of foundational legitimacy was confronted directly and at the highest levels of the state. Under Račan, such challenges to foundational legitimacy continued. The Croatian government undertook many vital military reforms, including a reduction of the bloated officer corps, cutting the total number of troops, investigating fraudulent disability claims, and other policies which were interpreted by veterans as a loss of the privileges they had under Tuđman’s HDZ.^18^

Combined with the indictments issued by the ICTY, the right-wing opposition viewed Račan’s downsizing of the military as an attack on the bedrock of the Croatian state. The willingness of veterans to challenge Croatia’s international obligations to cooperate with the ICTY became evident with the arrival of the first indictment for a Croatian officer in February 2001. The ICTY prosecution accused General Mirko Norac of crimes against Serb civilians in Gospić in 1991, but before he could be arrested he went into hiding. As many as 150,000 people took to the streets in the coastal city of Split to protest sending Norac to The Hague, a clear signal that the Croatian victory in the Homeland War was considered to be sacred.^19^ Norac eventually surrendered after Račan guaranteed that the case would be handled by a domestic court. But the mobilization potential of veterans was evident, and right-wing parties, including the HDZ, were willing to take advantage of it when they found themselves in the opposition. When the elderly General Janko Bobetko was indicted in 2002 for war crimes in the Medak Pocket operation, veterans guarded his house and threatened violence if Račan tried to forcibly arrest him. He died of natural causes before the indictment could be served, saving the government of a potentially dangerous crisis. The case of General Ante Gotovina, perhaps the most beloved hero of the Homeland War, dragged on for four years. The indictment for Gotovina and another Croatian officer, Rahim Ademi, had been unsealed in the summer of 2001, and while Ademi immediately surrendered with little protest, Gotovina went into hiding until finally being arrested on the Canary Islands in December 2005. Initially overshadowed by the crisis over Bobetko, Gotovina’s evasion of the ICTY became Croatia’s biggest foreign policy problem for years and delayed the country’s accession into the EU. More importantly, it indicated that Gotovina had a vast support network of retired and active *branitelji* who were willing to flaunt domestic and international laws to hide and protect him, as well as transforming him into a powerful political symbol that dominated the Croatian landscape during his time on the lam.^20^ The majority of protestors not only argued that Gotovina was innocent, but that he should not have to go to trial at all. This position corresponded with the statements of Milan Vuković, Croatia’s Chief Justice under Tuđman, who had infamously said that was not possible for the Croatian side to have committed war crimes because Croats had waged a defensive war.^21^

A network of veterans’ groups formed the backbone of the protest movement. Although the Organisation of Croatian Volunteers of the Homeland War (UHDDR – *Udruga hrvatskih dragovoljaca Domovinskog rata*) is the largest (approximately 300,000 members), the Croatian Military Invalids of the Homeland War (HVIDR-a – *Hrvatski vojni invalidi Domovinskog rata*) was the most vocal and aggressive veteran group in the debate over cooperation with the ICTY. There are dozens of other veteran organizations at the national, regional, and local level, and while they did not always have a unified position, they coordinated their actions under umbrella organizations such as the Headquarters for the Defense of the Dignity of the Homeland War (*Stožer za zaštitu digniteta Domovinskog rat*a), which was renamed in December 2005 to the Headquarters for the Truth of the Homeland War (*Stožer za istinu o Domovinskom ratu*).^22^ As an opposition party, the HDZ eagerly supported the massive protests of veterans; for example, Ivo Sanader was one of the speakers at the Split demonstrations against handing over Norac in 2001. However, once Sanader became prime minister in December 2003, he quickly changed his party’s rhetoric, supported full cooperation with the ICTY, facilitated the transfer of other Croats indicted by the Tribunal, and initiated an action plan that led to Gotovina’s arrest. Sanader successfully reined in the most radical veterans’ groups, and made important conciliatory moves towards the Croatian Serb community, antifascists, and civil society. The EU’s refusal to budge on Croatia’s obligations to cooperate with the ICTY, which could have potentially blocked accession indefinitely, explains why the HDZ under Sanader was willing to comply with the demands to arrest, try, and extradite Croatian officers. This did not indicate a change in the HDZ’s de-politicization of the veteran population, but rather a temporary compromise in the years immediately prior to Croatia’s EU membership. Sanader’s fall from power due to charges of corruption left his successor, Jadranka Kosor, in a weak position, allowing a reinvigorated SDP to win the 2011 elections in a campaign relatively bereft of ideological issues.

In December 2011, Zoran Milanović (SDP) became prime minister of Croatia with a strong mandate to extract the country from the devastating economic recession and corruption scandals of the previous government. On the doorstep of EU membership, it seemed that Croatia had overcome the burdens of its traumatic twentieth century history and was ready to seriously tackle the socio-economic challenges facing the entire region. The acquittal of generals Gotovina and Markač in the appeals chamber of the ICTY in November 2012 seemed to close a chapter on Croatia’s tangled history with the Tribunal and appeased the veterans’ groups, whose anti-ICTY stance had simmered even with the HDZ in power. The end of all trials at The Hague concerning Croats from Croatia with no guilty judgments appeared to pacify the right-wing political scene, which had used the war crimes issue to mobilize its supporters effectively for over a decade. Even Gotovina’s statement upon being released from the Hague in which he emphasized that Croatia needed to turn to the future hinted that the difficult past would be an issue handled by historians, educators, and civil society, and no longer subject to short-term political manipulations. Two seemingly minor events in 2012, however, would open the door for a new wave of symbolic politics: firstly, in April the *Sabor* ended its funding for the Bleiburg commemoration,^23^ giving the right-wing ammunition for its claims that the SDP was covering up communist crimes; and secondly, in May, Tomislav Karamarko replaced Kosor as chairman of the HDZ, resulting in a rapid shift towards a more nationalist, anti-communist direction for the party.

The first major mobilization of veterans after the end of the ICTY trials took place in 2013 as the government attempted to activate the law allowing for the use of minority languages and scripts on public buildings in Vukovar. Croatia’s regulations regarding minorities states that municipalities in which at least 33 percent of the population is a recognized minority, their language and script has equal parity with Croatian and Latin script in official use. In the case of Vukovar, this was Serbian written in Cyrillic for the Serb minority that had surpassed the minimum percentage in the 2011 census. The regulation had actually been established by the HDZ when it had been in power and in a coalition with the leading Croatian Serb party, the Independent Democratic Serb Party (SDSS). However, when the SDP-led government began installing Cyrillic signs on public buildings in Vukovar, it provoked an immediate backlash among veteran groups who argued that the city was a sacred place due to its suffering in 1991. In the right-wing discourse, Cyrillic was associated with the ‘script of the aggressor’.^24^ Following the model of the earlier anti-ICTY protests, veterans’ organizations formed a Headquarters for the Defense of Croatian Vukovar (*Stožer za obranu hrvatskog Vukovara*), which mobilized other groups throughout Croatia. In April 2013, this organization held a large protest in Zagreb, and when local authorities, protected by riot police, attempted to place the signs on buildings, masses of enraged veterans attacked the signs with hammers.

Even though the Constitutional Act on the Rights of National Minorities in the Republic of Croatia (*Ustavni zakon o pravima nacionalnih manjina*) enacted in 2002 guarantees the right to use Cyrillic, veterans argued that Vukovar’s sacredness transcended the rule of law: in other words, that foundational legitimacy trumped constitutional legitimacy. During the annual Vukovar commemoration on 18 November 2013, veterans’ organizations physically prevented Croatian politicians and the diplomatic corps from joining the Procession of Memory (*kolona sjećanja*) from the Vukovar hospital to the memorial cemetery, forcing them to abandon the official ceremony in a scandal of national proportion. The HDZ, in the opposition and still reeling from the corruption affairs, found an emotional cause around which to build a symbolic political platform, accusing the SDP government of being ‘against the people’ and anti-veteran. Although denying any connection with the protests, Karamarko, himself a *branitelj* due to his work in the police force, could be seen in the company of the protest organizers, clearly pleased at the weakness of the government in implementing the rule of law when faced with determined veterans. The veterans also organized a referendum to try and change the minority law so that the minimum percentage of minorities in a municipality be raised to 50 per cent in order to be allowed to use their own language and script, but the Constitutional Court ruled this unconstitutional in August 2014. The government was forced to back down, and the issue continued to seethe until a HDZ mayor took over in Vukovar and changed the city statute to declare the town a place of special piety.

Meanwhile, a new protest erupted, representing the most serious threat to Croatia’s legal institutions since independence. On 20 October 2014, veterans began camping in front of the Ministry of Veteran Affairs in Zagreb, setting off a protest that would last 555 days. The veterans’ initial demand was the resignation of Minister Predrag Fred Matić, a veteran of Vukovar and survivor of Serbian internment camps, and his assistant Bojan Glavašević, the son of legendary Vukovar reporter Siniša Glavašević, who was killed in the Ovčara massacre.^25^ They were accused of ‘equating victims and aggressors’ in a statement about the number of *branitelji* suffering from post-traumatic stress disorder, as well as plans by the ministry to increase support for civilian victims of the war.^26^ On 21 October, a disabled female veteran, Nevenka Topalušić, died during the protest due to poor health, which spurred on the veterans even more. A number of tents were erected in front of the ministry as the veteran groups settled in for the long haul, leading the media to dub the event the Tent Protest and the veterans as ‘tentmen’ (*šatoraši*).

Other than the resignation of Matić and Glavašević, the other demands of the *šatoraši* were unclear and changed as the protest dragged on. An oft-repeated demand was for the laws regulating veterans’ statuses to be enshrined in a single constitutional law, which would make it difficult for any future government to alter their privileges and benefits. Many veterans were also angry at the ministry for making the Registry of Veterans available to the public, which was intended to make transparent the list of who had received veteran status. Some right-wing groups called for the creation of a Registry of Aggressors, potentially increasing the sense of insecurity among Croatia’s remaining Serb population, already regularly blamed for the war and all of Croatia’s post-war problems.^27^ The veterans on the street, at times physically threatening the employees of the ministry, believed they had the right to change the legally elected government through pressure and scare tactics. While the Croatian government and city of Zagreb are generally strict in issuing permits for setting up tents and other structures, as well as protests, no permits were ever issued for the tents that stood in front of the ministry for over a year. Despite the clear violations of the rule of law, HDZ politicians, including the newly-elected President Kolinda Grabar-Kitarović, regularly visited the veterans in the tent. Karamarko, when asked to comment on whether protesting veterans had broken the law during a scuffle with the police in front of the Parliament building, told reporters: ‘What law? These are *branitelji*. Are we going to treat *branitelji* in wheelchairs like hooligans who break the law?’^28^ Dissident voices among the veteran population were drowned out by the more organized *šatoraši* receiving thinly veiled support from the HDZ.

The Tent Protest was framed as a continuation of the Homeland War, with many banners, signs, and statements referring to a new struggle against communists and Yugoslavs. Under the motto ‘100% for Croatia’, the protestors claimed ‘We created it [Croatia], the politicians are destroying it.’^29^ Prime Minister Milanović was regularly criticized for not being a *branitelj*, and the question of ‘where were you in 1991?’ took aim at one’s loyalty to the post-1991 Croatian state. At numerous press conferences and gatherings organized on the main square, protest leaders claimed that they had the exclusive right over Croatian history, politics, and economic policies, suggesting that their sacrifices in the 1990s were not adequately rewarded. The statements increasingly began to resemble those of SUBNOR, the war veterans’ association in communist Yugoslavia, although the phrases did not refer to the nurturing of the revolution but rather the preservation of the values of the Homeland War and the mythical golden age of Croatian unity that supposedly existed in that period. As in the case of SUBNOR, many *branitelji* did in fact suffer from PTSD, poverty, and health problems, while the leaders of the veteran organizations lived quite well off from their privileges and political connections.^30^

The elections of November 2015 resulted in the formation of a coalition government between the HDZ and a newly formed third party, MOST (Bridge). Karamarko had engaged in highly ideologically charged campaign, regarding both the Second World War (specifically referring to the end of funding for the Bleiburg commemoration and calling for a lustration of allegedly embedded communist structures) and the Homeland War. He presented the HDZ as the true defender of the *branitelji* and the culture of victory, while accusing Milanović of not taking a patriotic enough stance on relations with Serbia and Bosnia-Herzegovina. After a close election with no clear winner, Tihomir Orešković, a Canadian-Croatian businessman was brought in as a non-party prime minister, and Tomo Medved (HDZ) was appointed to head the Ministry of Veteran Affairs.^31^ With the key demand of the Tent Protestors satisfied, the veterans dismantled the tents in May 2016. Although Orešković’s government fell after a series of scandals that paralyzed the functioning of the administration, it was replaced by a new HDZ-led coalition under Prime Minister Andrej Plenković. With the HDZ back in power, organized veterans’ protests came to an end.

Public perception for much of the time of the Tent Protest is that *branitelji* live privileged lives, while in fact many face poverty, health problems, and a lack of public recognition. The silent majority have moved on with their lives and are not active in protests or challenges to the legal system. However, it is clear that the veteran organizations play a significant role in Croatian politics, and the leadership has benefitted from the culture of victory in the country.^32^ According to one estimate, approximately 1350 veteran organizations are active in Croatia, and investigative reporting by *Jutarnji list* noted that in 2016 they received nearly 50 million kuna (6.7 million Euros) from the Ministry of Veteran Affairs, other state funds, and municipal budgets.^33^ The four biggest veteran organizations are led by individuals – Đuro Dečak, Tomislav Merčep, Josip Đakić, and Zvonko Milas – who are HDZ deputies or former HDZ functionaries, pointing to the close relationship between two organizational champions of foundational legitimacy. Ante Deur, one of the leaders of the Tent Protest, is an advisor to President Grabar-Kitarović. During SDP governments, any kind of crisis or international dispute, especially regarding territory or relations with Serbia, provoked immediate reactions from veteran organizations. Since the HDZ has been in power, a loss of territory to Slovenia in an international arbitration, provocative statements from Serbia, and a debilitating scandal regarding corruption in Croatia’s biggest company, Agrokor, the previously vocal veteran organizations have remained obediently silent. The new benefits prepared by Minister Medved certainly contribute to the sense of satisfaction, since *branitelji* pensions have increased, access to jobs and education have been expanded, and a new proposal to require all municipalities to increase spending on veterans’ organizations is in the works.

But there is no doubt that the well-organized network of veteran organizations would be ready for mobilization in case the HDZ found itself once again in opposition, regardless of the conditions that would lead to such a situation. This appears to be an army not for fighting off external threats, but an internal war for those questioning the system established through appeals to foundational legitimacy. Veteran HDZ politician Vladimir Šeks identified precisely that war in statements in an interview in 2011:

No other nation in history has paid such a heavy price for its freedom as the Croatian people. Today Croatia is a free, independent, and democratic country, but it has not yet achieved its goals. The Homeland War, in which the Croatian people battled for their freedom, was a three-part war for Croatia – a war with the Serbian aggressor, a war with the international deniers of Croatian independence, and a war with domestic revisionists and falsifiers of Croatia’s path to statehood, independence, and victory in the imposed, justified, defensive, and liberating Homeland War. The first war was waged with weapons, the second with diplomacy, and the third with a promotional campaign. The Croatian people won the first two wars, but the third is still being fought. It is up to us to finish it as we did the first two – with victory.^34^

The appeal to foundational legitimacy by certain memory entrepreneurs who often speak in the name of the entire veteran population does not imply that the contribution of Croatian war veterans to establishing a democratic, independent state should be seen as simply a grab for political and economic power. However, political forces, primarily on the right-wing political spectrum, have used the foundational legitimacy of participation in the Homeland War as a justification for subverting the rule of law when it comes to identity politics, fighting corruption, or ensuring transparency at all levels of government.^35^

In February 2018, Kosovo celebrated the tenth anniversary of its declaration of independence. Nearly a year before that, the tiny country held its third post-independence elections, which like previous elections was a contest between entrenched political parties and elites keen to hold on to their control over state institutions, important sources of graft. The PDK, which President Hashim Thaçi once lead before resigning to become head of state, joined forces with former Prime Minister Ramush Haradinaj's AAK and Fatmir Limaj's Initiative for Kosovo (*Nisma për Kosovën*), forming a coalition called ‘PAN’. Each of these groupings had roots in the former UÇK guerilla movement. As one analyst noted, ‘if parties of the so-called PAN coalition had anything in common, then it is they represent a political class driven by personal gains and benefits, rather than being driven by principles and ideas of governance.’^36^ French press agency AFP referred to the election results as a ‘coalition of warriors,’ which was widely quoted in the regional media.^37^ The 39-page electoral program (apparently full of spelling mistakes) prepared by coalition leader PDK included a museum commemorating Kosovo’s liberation among the campaign promises, part of a well-trodden effort to emphasize their foundational legitimacy. But there was something else that may have united these political groups. What distinguished the 2017 contest from previous elections, as Capussela notes, was that the politicians behind it are mostly former UÇK commanders who hope that being in power will better protect them from indictment and prosecution by a special tribunal set up to try Kosovars for crimes committed during the 1999 war of independence.^38^ Just as the European Union sees dealing with this dark past as a necessary prerequisite to eventual membership for Kosovo, so do ex-UÇK commanders see it is as a direct threat to the position and privileges they have established over the past nearly two decades.^39^

The struggle for ethnic Albanian rights in Kosovo dates back to the first Yugoslavia,^40^ continued during the Second World War and its aftermath as well as during socialist Yugoslavia, when domestic and diaspora-based Albanian groups led movements for recognition of Albanians as a nation within Yugoslavia.^41^ Some Kosovar Albanians dreamt of unification with Albania, which until 1991 was led by a regime that was simultaneously communist and nationalist. A Kosovar independence movement emerged after the collapse of socialist Yugoslavia in the 1990s, when the Serbian regime implemented apartheid-like policies in Kosovo, denying ethnic Albanians, among other things, the right to secondary and tertiary education. Resistance to Serbian rule soon appeared. Led by intellectual Ibrahim Rugova, ethnic Albanians in Kosovo organized themselves around a new political party, the Democratic League of Kosovo (*Lidhja Demokratike e Kosovës*, LDK), and began to establish parallel institutions: health, welfare, and education, among others. The LDK called for peaceful resistance and negotiations with the Belgrade-based authorities. Throughout the twentieth century, Albanian resistance movements in Kosovo had been divided between those advocating peaceful means and those pushing for armed rebellion. In the second half of the 1990s, the armed resistance evolved out of frustrations with the peaceful approach of the LDK (which some militants labeled ‘morbid pacifism’)^42^ and its lack of results, as well as intensifying repression by Belgrade. Some Kosovars had lobbied the West to intervene, but the international community was consumed with the wars in Croatia and Bosnia-Herzegovina. After the Dayton Peace Accord of 1995, there was ‘Balkan fatigue’ among Western policymakers, and Serbian strongman Milošević came to be seen as a peacemaker. Kosovars were left to their own devices.

It was in these conditions that the armed resistance took shape. In the early days of the insurgency, the UÇK was advised by the Popular Movement of Kosovo (*Lëvizja Popullore e Kosovës,* LPK), a long-standing opposition group made up mostly of exiled Kosovar Albanians. It acted as a political wing, advising rebel commanders on how to sell themselves to an international audience and to respect the international conventions. However, this relationship frayed over time, as LPK leaders, who mostly lived in the West, found it hard to connect to rebel commanders on the ground in Kosovo. Other armed cells sprung up at this time, but the UÇK soon became the dominant force and absorbed many of their fighters, but not all.

The exiled LPK deployed another militia, the Armed Forces of the Republic of Kosovo (*Forcat e Armatosura e Republikës së Kosovës,* FARK). The leaders of the UÇK and FARK failed to reach an agreement on cooperation in mid-1998, and the FARK refused to hand the UÇK funds collected from the Kosovar diaspora. FARK was subsequently accused of treason by the UÇK and fighting broke out between the two rival troops in some parts of Kosovo, foreshadowing the symbolic politics of victory that took shape after liberation.^43^

As violence increased, the international, and particular US, response at first appeared to suggest opposition to the UÇK. Perhaps driven by a perception of external support, Serbian security forces carried out a ruthless campaign against the armed insurgency. On 5 March 1998, Serbian forces slaughtered 56 members of the extended Jashari clan in the village of Prekaz, along with the famed UÇK commander Adem Jashari. Jashari was later lionized by the UÇK and its successor parties as a ‘legendary commander’ (*komandati legjendar*), and his name is invoked frequently as a symbol of sacrifice and patriotism in the mold of early twentieth-century Albanian resistance fighters. According to the scholars Di Lellio and Schwander-Sievers, Jashari provided a ‘powerful counter-narrative to the one of victimization and accommodation with the enemy’.^44^ In the long term, he also provided the UÇK and its offshoot parties with an important ingredient in their claims of foundational legitimacy.^45^ The massacre at Prekaz further helped to galvanize support for the UÇK and armed resistance, and tens of thousands of Kosovar men enlisted. As international diplomatic interest in the conflict increased, the UÇK created a political directorate which acted as a messaging and diplomatic arm for the armed resistance, with Hashim Thaçi, then a young commander, at its helm. These were the roots of the PDK.

After the NATO intervention and Rambouillet Peace Agreement of 1999, the victorious UÇK was disarmed and demobilized fighters joined the police, newly-formed civilian protection corps, and private security agencies. Top commanders, for their part, capitalized on the UÇK's popularity and quickly went into politics. The UÇK and its role in the liberation from Serbian oppression quickly became one of the main symbolic features of the newly-liberated Kosovo.^46^ The UÇK was seen by most of the Kosovar Albanian population as the army of a future Kosovar state, and there was fierce opposition to any plans that envisioned its complete elimination. As a result of such popular support, occupying NATO forces allowed it to remobilize in the form of the Kosovo Protection Corps (*Trupat e Mbrojtjes së Kosovës,* TMK), whose organization and structure mirrored that of the UÇK. Attempts to include ethnic Serbs in its ranks were vigorously opposed by ethnic Albanians.^47^ Moreover, ethnic Serbs deeply distrusted the TMK, seeing it as a UÇK in new clothes.

The PDK was formally established in September 1999, with Thaçi at its head. A separate party, the AAK, was launched by Ramush Haradinaj, who commanded UÇK forces in western Kosovo. Rather than being divided by any discernible ideological or programmatic differences, the two parties became quickly associated with the personalities that led them, and they tended to attract adherents from their respective regions. While the LDK, the pacifist party of former president Ibrahim Rugova, continued to play a role on the political scene after 1999, it was forced into coalitions with the two UÇK parties. From the beginning, the PDK and AAK invoked the UÇK legacy to seek support and fight critics as they built networks of corruption, patronage, and criminality. Yet, they have also competed over the right to be regarded as the authentic heirs of the Kosovo Liberation War.

Following Kosovo’s internationally-guided declaration of independence in 2008, the TMK was dissolved and replaced by a new structure, the Kosovo Security Force (*Forcat e Armatosura të Kosovës,* FAK). Although the FAK successfully recruited many former UÇK/TMK members, the TMK’s dissolution was still seen as a blow to the UÇK’s legacy, and was fiercely opposed by UÇK-linked politicians.^48^ But after seven years, the trappings of political power were even more attractive, and all Kosovar politicians understood that they served at the behest of Kosovo’s international supervisors. Consequently, they often fell into line with the demands of the international community to refrain from mistreating the remaining ethnic Serb residents of Kosovo, as well as adopting a host of other policies mandated by officials of the international protectorate. Given the billions of dollars in financial, legal, and economic assistance flowing into the tiny country, it was in the interest of the newly-emerging Kosovar political class to cooperate with the ‘internationals’. Meanwhile, the international institutions that helped administer Kosovo after 1999, such as UNMIK (United Nations Mission in Kosovo), the OSCE (Organisation for Security and Cooperation in Europe), and KFOR (Kosovo Force), were in turn dependent on the locals to succeed. The nascent Kosovar political elite owed its success to close relationships with international actors. Thaçi, who first served as prime minister and later as president, became the ‘go-to guy’ for US, European, and UN officials seeking to show progress in Kosovo. Accordingly, his control over Kosovo’s political and economic life grew, and he began to eliminate political alternatives through co-optation, control of the media, and at times intimidation.

Yet, some scholars have pointed out that the presence of a strong colonial-like international administration did not preclude tension between external actors and veterans’ groups espousing foundational legitimacy:

The local actors of UÇK veterans’ circles sought to establish an Albanian nation-state, in which the veterans were to be rewarded with a prominent role as liberators. In contrast, the international administration endeavoured to forge a multi-ethnic society within the framework of the international peacekeeping mission.^49^

By the second decade of Kosovo’s transition from Serbian rule, Kosovo’s leading politicians and parties – the PDK and Haradinaj’s AAK – had successfully converted their wartime exploits in to a vast patronage network. In 2016, Transparency International, the corruption watchdog, ranked Kosovo 95th out of 176 countries in terms of perceptions of corruption, the worst result in the Balkan region.^50^ According to the findings of a Council of Europe investigation led by the former Swiss prosecutor Dick Marty, Thaçi and his allies had amassed staggering wealth far beyond their public salaries through corruption and organized crime. The investigation alleges that Thaçi was a feared figure during the conflict, ruthlessly eliminating potential rivals.^51^ The Council of Europe Report focused on the activities of the so-called ‘Drenica Group’, allegedly consisting of top UÇK commanders who later became PDK officials, including Thaçi, Kosovo Assembly chairman and current PDK leader, Kadri Veseli, and other senior PDK figures.^52^ The alleged crimes include killings, abductions, organ harvesting, illegal detentions, drug trafficking and sexual violence during and after the 1998–9 war.^53^ Earlier reports corroborated such abuses. For example, in 2009, Amnesty International reported that some 800 murders were committed in the period after the withdrawal of Serb forces in 1999, with the majority of victims Serbs or Kosovo Albanians labelled as collaborators with Serbia.^54^ In 2013, a US publication, *The New Yorker,* published an extensive reportage on Thaçi’s alleged involvement in abuses and crimes such as organ trafficking.^55^

In many cases, the targets of UÇK crimes were members of Rugova’s more moderate LDK movement. A number of Rugova’s close associates were murdered or wounded in attacks during the war. Ahmet Krasniqi, defence minister in Rugova’s government in exile, was also shot dead in Tirana in 1998. There was an attempted murder against LDK presidency member Sabri Hamiti in 1998 and Rugova’s close friend and ally and the LDK’s head of public information, Enver Maloku, was killed in 1999. Other LDK officials who were members of Kosovo’s parallel parliament under Serbian rule, including former president Fatmir Sejdiu, were detained by the UÇK in the Drenica Valley. One of them, Gjergj Dedaj, has claimed that the detainees were personally interrogated by Thaçi and that they were tortured as well.^56^ After the war, several senior UÇK members loyal to Rugova and some of his closest allies were then killed or attempts were made on their lives: Ekrem Rexha, Tahir Zemaj, Smajl Hajdaraj, Shaban Manaj, and Rugova’s closest adviser, Xhemajl Mustafa. Fetah Rudi, the former head of the LDK’s branch in Malisheva/Mališevo, was also shot immediately after the 2000 local elections and was paralyzed. As many as 100 LDK activists, officials and prominent supporters were abducted, murdered, or wounded between 1998 and 2001, and survivors and relatives accuse the UÇK of carrying out these crimes.^57^ Remarkably, owing to political expediency, a culture of clientelism, and Kosovo’s electoral system, until 2017 the LDK was in coalition with the PDK three times.

Thaçi answered the accusations against him and the UÇK by accusing the Council of Europe and other critics of defaming the UÇK and the memory of the fighters who gave their lives for Kosovo’s independence. Meanwhile, other former UÇK fighters have remained active in Kosovo’s criminal underworld, engaged in activities such as drug running and human trafficking.^58^ Yet, the international community, while spending millions on rule of law programs, has been unable to root out the endemic corruption. Some allege that the EU’s rule of law monitoring body, EULEX, has even been complicit in the corruption. Thaçi has also been shrewd in making himself ‘indispensable’ to internationals, as a negotiator with former foes in Serbia (which some argue only a former UÇK fighter had the necessary political cover to engage in) and most recently as an ally in the fight against the Islamic State (hundreds of Kosovars have traveled to Syria and joined IS). Thaçi has even appealed to the West by publically supporting LGBTQ rights. In his effort to remake himself as a liberal democratic politician, he has succeeded in winning over a number of Western politicians, among them former US Secretary of State Madeline Albright and former Vice President Joe Biden. The latter once called Thaçi the ‘George Washington of Kosovo.’^59^

Former UÇK figures have also used their status to engage in other crimes. Azem Syla, a prominent legislator from the PDK, was accused by the EU rule of law mission in 2016 of being the alleged ringleader of an organized crime group which engaged in money laundering, expropriation of public and ethnic Serb property, forging of official documents, corruption, aggravated fraud, fraud in office, unlawful court decisions, abuse of office, legalization of false assets, fiscal evasion, and money laundering.^60^ ‘A structured criminal group with a long-term, organized hierarchy … damaged Kosovo's state budget and Kosovo Serbian families whose land rights were abused,’ the prosecution said. The group allegedly had ties with Serbian criminals as well. Two other former judicial officials, Nuhi Kuka and Safedin Haxhiu, were also implicated in the indictment. Syla was released in March 2018, after the case was transferred from international to local judges.^61^

One of the greatest challenges to the UÇK’s claims to foundational legitimacy came, as in the case of Croatia, from a tribunal, in this case the Kosovo Specialist Chambers, which the Kosovar parliament blessed under enormous external pressure in 2015. Consisting of foreign staff, judges, and prosecutors, its work is based on long-standing accusations against the UÇK contained in the aforementioned Council of Europe report – for which there is apparently substantial evidence – that senior UÇK members (perhaps including Thaçi and others) engaged in unlawful killing, abduction, illegal detention, sexual violence, forced displacement, and illegal organ harvesting during the conflict against Serbia.^62^ These charges, more than anything, constitute a threat to not only Thaçi, but the entire UÇK leadership, who owe their positions to foundational legitimacy. Halil Matoshi, a Prishtina-based political analyst, has written:

If this court truly becomes functional and if it is guided by evidence and not by politics, and if it has sympathy for the victims and the will to do justice, then it would profoundly influence in Kosovo’s political scene, emphasizing new values instead of the old ones - arrogance, aggression, ethnic cleansing and other people’s suffering as patriotic and heroic aims.^63^

Thaçi has responded to such threats using the defense that has served him so well in the past: ‘I was fighting on the right side of history, liberating my people from tyranny against a ruthless enemy engaged in a massive attempt at genocide,’ he told the *New York Times*.^64^ Following a visit of Specialist Prosecutor Jack Smith to Pristina in October 2018, Prime Minister Ramush Haradinaj told media that while ‘Kosovo indeed has obligations towards the Specialist Chambers … there is also a sense of scepticism about these processes involving international justice’.^65^

The political machinations discussed at the outset of this section are culminations of a trend in which the Kosovar political class, led by former liberation fighters, has held the reins of power, captured critical state institutions such as law enforcement and the judiciary. The ruling parties also control the media^66^ and dole out jobs and other forms of patronage to political supporters. Meanwhile, average Kosovars suffer from massive levels of unemployment (youth unemployment stands at 50 per cent) and associated poverty rates.^67^ The average monthly wage in Kosovo is just €360, and the economy is highly dependent on remittances.^68^ Tens of thousands of Kosovars were among the enormous waves of migrants attempting to enter Germany and other EU countries in 2015.^69^ While in Croatia the HDZ and veterans’ groups have openly played the nationalist card for political gain, the PDK and other UÇK-derived groups, under close international supervision, have had to tone down such rhetoric – for the most part. But they have undermined democratization, reconciliation, and minority inclusion in other ways. In 2017, PDK-majority parliament, for instance, took a nationalist line and failed to pass critical legislation demarcating the border with Montenegro and establishing an Association of Serb Municipalities within Kosovo. It later voted to ratify the border deal.^70^ In another sign of a growing nationalist line in Pristina, in early November 2018, the Kosovo government decided to impose a ten percent tariff on goods from Serbia and Bosnia, apparently in violation of the Central European Free Trade Agreement (CEFTA).^71^ In September 2018, thousands of Kosovars protested against a proposed land swap with Serbia.^72^ In part, the Kosovar ruling parties are under pressure from an emboldened nationalist opposition, *Vetevendosje* (Self-Determination), but they are also keen to overcome their own limited legitimacy by taking a nationalist line.

And yet, much like Croatia, UÇK-affilated veterans’ organizations have also played a leading role in upholding a culture of victory in Kosovo. Isabel Ströhle identifies a number of organizations who have fought to defend ‘war values’ (*vlerat e luftës*), such as the Organisation of the UÇK War Veterans (OVL-UÇK), the Association of the UÇK Invalids (SHIL) and the Association of the UÇK Martyrs’ Families (SHFD). They have continually lobbied for greater long-term social protections and benefits, which not only clashed with the plans of international administrators, whose focus was on short-term reintegration of former fighters, but more generally with the multi-ethnic, liberal vision of a future Kosovar state that the ‘internationals’ espoused.^73^ In the late 2000s, veterans organized protests and hunger strikes to press for greater benefits, while some UÇK-affiliated politicians pushed for a law that would recognize Yugoslav-era resistance fighters as martyrs (*dëshmorët*) as well (thereby further stigmatizing association with the former Yugoslav state).^74^ Unsurprisingly, members of other armed groups, like those affiliated with the LDK, were often excluded from such initiatives.

A former UÇK commander-turned-politician, Fatmir Limaj, best expressed the sentiment of the veterans’ organizations in his response to the UN administration’s proposed changes to a law governing the status of veterans:

We cannot accept that somebody should play with our past as it suits them and that some- body should play with terminology based on how others interpret our war. This law has been made for the citizens of Kosovo and will be applied to its citizens. For the citizens of Kosovo there has been a war of liberation, for the citizens of Kosovo there were occupiers and enemies and for the citizens of Kosovo there have been and there will be, martyrs and heroes of the people. This is a historical law and we will not allow the administrators to write our national history … In Kosovo, nobody of whatever leaning ever was able to change our history and even less so with administrative instructions.^75^

Yet, unlike Croatia, where possible challengers to constitutional legitimacy – the generals fired by Mesić in 2000 – were sidelined from formal power in the second decade of transition, in Kosovo former paramilitary figures lead the state. Thus, in Kosovo, the culture of victory has meant state capture by a group of former rebel fighters who converted their liberation role into political and economic power. If the power of foundational legitimacy in Croatia is found in the ongoing nationalist narrative of the Homeland War, then in Kosovo it can be located primarily in the perpetuation of foundational legitimacy through political parties, patronage, corruption, and criminality.

In both Croatia and Kosovo, liberation was achieved by men with guns who subsequently converted their hard power into the soft power of a hegemonic and resilient foundational narrative that has allowed them to sustain political and economic power while covering up corruption and crimes.^76^ Using a culture of victory, the HDZ in Croatia and the UÇK/PDK in Kosovo instrumentalized foundational legitimacy and converted their role in the independence struggle into dominant post-independence political and economic roles. Foundational legitimacy, in other words, allowed political parties and affiliated veterans’ organizations in both countries to sideline challengers by wielding the sword of foundational legitimacy.^77^ Among the victims of cultures of victory in Croatia and Kosovo have been a free media, ethnic minority rights, and open debate. And in the worst cases, foundational legitimacy has challenged constitutional legitimacy itself.

In both Kosovo and Croatia, the politicization of the liberation struggles and the active role of former fighters in everyday politics makes it clear that they are far from simple veterans, especially if compared to the invisibility of veteran groups in Serbia or Bosnia-Herzegovina. In these countries, veterans’ groups are marginalized and divided, with relatively little influence unless directly involved in a political party. One explanation is that these two countries lack the culture of victory as displayed in Croatia and Kosovo, which has elevated veterans to their current positions in society. Serbia has an ambivalent position to its wartime past, since officially Belgrade denied its role in the conflicts of the 1990s, while in Bosnia-Herzegovina a fractured memory of the war prevents the creation of a single narrative.

In both countries, the culture of victory is regularly performed at numerous commemorative events throughout the year. These commemorations serve to highlight the role of liberation fighters, as well as stifle critical interpretations of the conflict. In Croatia, we have seen the HDZ encouraging and tacitly supporting veterans’ groups and their mass protests when the party was in opposition, and reining in the veterans when in power. Peaceful demonstrations are legitimate forms of protests in democratic societies, but in the Croatian case the *branitelji* brought a militarized discourse to the streets with barely veiled threats against perceived enemies of the state. At a public roundtable in the midst of the Tent Protest, philosopher and human rights activist Žarko Puhovski posed the question ‘why are there still *branitelji* (defenders) and not just veterans, since the country no longer needs to be defended?’^78^ In Kosovo, politicians regularly gather to commemorate the sacrifices of the Jashari clan as a symbol of the liberation battle, and veterans’ organizations have also put on protests to push for greater recognition and privileges.

However, in Croatia, there have been meaningful challenges to the culture of victory, from internal political and social actors willing to question the unconditionally heroic narrative to international trials exposing the Croatian state’s knowledge of war crimes perpetrated domestically and in neighboring Bosnia and Herzegovina. In Kosovo, by contrast, a culture of victory and the politics of foundational legitimacy have never been successfully challenged, even as their expression is often not as explicit as in Croatia. Because the main champions of foundational legitimacy in Kosovo have been under close international supervision, they have learned to speak the language of democracy, human rights, and inclusion. The internationals, for their part, have ignored the wrongdoings of top politicians to achieve their own ends, such as extracting concessions to Serbia and showing progress on transition. Nevertheless, former UÇK commanders-turned-politicians successfully used foundational legitimacy to entrench themselves in corrupt patronage networks. Corruption, nepotism and lack of proper governance have in turn also stalled Kosovo’s economic and social development and held back full democratization. The Kosovo Specialist Chambers’ indictments, whenever they come, are likely to pose the most serious challenge to the UÇK’s foundational legitimacy narrative thus far, and Kosovo’s politicians are clearly fearful of what this may mean for their entrenched privileges.

Foundational legitimacy should not be confused with foundational myths. All states have powerful narratives about their genesis, and contested memories are common, even in the most developed democracies. The significance of foundational legitimacy lies in the way it is instrumentalized by actors in new states such as Kosovo and Croatia to bid for power and resources within the context of a culture of victory, and the way in which this has undermined liberalism. These trends can be seen in other cases of political actors in new states that use war victory to shore up their political legitimacy, putting war veterans on a pedestal, as the studies in this special edition covering the successor states of the First World War – Poland, Yugoslavia, and Czechoslovakia – demonstrate. In other cases, especially those in the postcolonial world, foundational legitimacy becomes embedded in state identity and ruling regimes, as it is in countries such as Zimbabwe or Algeria.

## Notes

1 See M.-R. Trouillot, *Silencing the Past: Power and the Production of History* (Boston, MA 1995); G. Cubitt, *History and Memory* (Manchester 2007); and T. Sindbaek, *Usable History? Representations of Yugoslavia’s Difficult Past from 1945–2002* (Aarhus 2012).

2 E. Jelin, *State Repression and the Struggle for Memories* (London 2003). Memory entrepreneurs, or mnemonic actors, can be individuals or groups that are influential in creating and reproducing historical narratives, either through bottom up initiatives (such as victims’ organizations, war veteran associations, or similar civil society groups) or through institutionalized memorialization (museums, schools, official commemorations, etc.).

3 See D. Pauković and V. Raos, ‘Democratic Deficits, Delayed Democratization and Prolonged EU Accession,’ in P. Maldini and D. Pauković (eds) *Croatia and the European Union: Changes and Development* (Farnham 2015); O. Lustig and Z. Strabac, ‘Support for Democracy and Strength of Civil Attitudes: Croatia Compared with New and Old Democracies,’ in S.P. Ramet and D. Matić (eds) *Democratic Transition in Croatia: Value Transformation, Education & Media* (College Station, TX 2007); and J.N. Clark, ‘Giving Peace a Chance: Croatia’s Branitelji and the Imperative of Reintegration,’ *Europe-Asia Studies*, 65, 10 (2013), 1931–53.

4 S. Durašković, ‘National identity-building and the “Ustaša-nostalgia” in Croatia: the past that will not pass,’ *Nationalities Papers*, 44, 5 (2016), 772–88.

5 D. Marijan, *Hrvatska 1989.–1992: Rađanje države* (Zagreb 2017).

6 *Branitelj* status is regulated by the ‘Law about Croatian Defenders from the Homeland War and the Members of their Families,’ initially passed in 2004 and most recently amended in December 2017, available at: https://narodne-novine.nn.hr/clanci/sluzbeni/2017_12_121_2758.html. (accessed 19 March 2019). Article 3 defines a *branitelj* as an ‘individual who participated in the organized defense of the independence, territorial integrity and sovereignty of the Republic of Croatia as a member of the Armed Forces of the Republic of Croatia’ or as an armed member of the *Narodna zaštita* (National Defense), initially created by the HDZ in 1991 as a civilian organization to assist the government at a time of rising tensions with the JNA.

7 K. Kardov, ‘Remember Vukovar: Memory, Sense of Place, and the National Tradition in Croatia,’ in Ramet and Matić (eds) *Democratic Transition in Croatia*, 82.

8 For estimates of civilian deaths and number of people who left the Krajina, see Gotovina et al. (IT-06-90), available at: http://www.icty.org/case/gotovina/4 (accessed 19 March 2019). Eastern Slavonia remained under Serb rule until peaceful reintegration into Croatia in 1998.

9 Ivo Goldstein, *Hrvatska povijest* (Zagreb 2003), 428.

10 M.P. Boduszyński, *Regime Change in the Yugoslav Successor States: Divergent Paths Toward a New Europe* (Baltimore, MD 2010).

11 Pauković and Raos, ‘Democratic Deficits’, 39–41.

12 The law is available at www.vlada.hr/Download/2002/12/12/Zakon.Suradnji.Haag.htm. (accessed 19 March 2019).

13 C. Lamont, *International Criminal Justice and the Politics of Compliance* (Farnham 2010), 48–51.

14 The full text is available at http://narodne-novine.nn.hr/clanci/sluzbeni/2000_04_41_957.html (accessed 19 March 2019).

15 V. Pavlaković, ‘Croatia, the ICTY, and General Gotovina as a Political Symbol,’ *Europe-Asia Studies* 62, 10 (2010); and V. Peskin and M.P. Boduszynski, *Croatia's Moments of Truth: The Domestic Politics of State Cooperation with the International Criminal Tribunal for the Former Yugoslavia* (Berkeley, CA 2003).

16 R. Lukić, ‘Civil-Military Relations in Croatia (1990–2005),’ in S.P. Ramet, K. Clewing and R. Lukić (eds) *Croatia since Independence: War, Politics, Society, Foreign Relations* (Munich 2008), 201–6.

17 DeUÇKracija o Domovinskom ratu, online version at *Narodne Novine* (13 October 2000), http://narodne-novine.nn.hr/clanci/sluzbeni/274008.html (accessed 19 March 2019).

18 T. Edmunds, *Defence Reform in Croatia and Serbia-Montenegro* (Oxford 2003), 37–44.

19 See Peskin and Boduszynski, *Croatia's Moments of Truth.*

20 V. Pavlaković, ‘Deifying the Defeated: Commemorating Bleiburg since 1990,’ *L’Europe en Formation*, 357 (2010).

21 *Vjesnik* (15 March 2004), 5.

22 *Novi list* (12 December 2005). In February 2006, the Foundation for the Truth of the Homeland War was established to raise money for Gotovina’s defense, fund research on the Homeland War, and provide scholarships to the children of veterans.

23 The Bleiburg commemoration, held annually in May in the small Austrian town of Bleiburg near the Slovenian border, commemorates the postwar repression of Ustaše and other alleged collaborators, including a number of civilians, by the victorious Partisans and newly established communist regime. The commemoration was used by emigre groups to challenge the Yugoslav regime prior to 1990, and since then has become an official, if controversial, commemoration due to the tendency to glorify the Ustaša movement rather than pay homage to the memory of communist repression. See Pavlaković, ‘Deifying the Defeated.’

24 For an analysis of the public discourse around the Cyrillic issue, see L. Šarić and T. Radanović Felberg, ‘Cyrillic does not Kill: Symbols, Identity and Memory in Croatian Public Discourse,’ *Družboslovne Razprave*, 33, 85 (2017), 51–71.

25 For a description of the crimes committed after the fall of Vukovar, see the ICTY judgement for ‘Vukovar Hospital’, at http://www.icty.org/x/cases/mrksic/tjug/en/070927_Mrksic_radic_Sljivancanin_ summary_en.pdf (accessed 19 March 2019).

26 At a press conference in Vukovar, Glavašević had provoked the veterans with the following statement: ‘How come Croatian veterans and civilian victims on the Croatian side suffer from numerous ailments and PTSD, and this is a population that won the war, and received numerous benefits and pensions. On the other side, we have a population from the former Krajina that lost the war, has no rights, and no PTSD. Perhaps we don’t see them because they aren’t in hospitals. This question has no answer yet, but perhaps we should find it, no?’ *Globus* (25 March 2016), 13.

27 The Serbian National Council (Srpsko narodno vijeće) regularly publishes reports of hate speech and discrimination against Serbs in Croatia. See T. Opačić, *Historic Revisionism, Hate Speech and Violence against Serbs in 2017: SNV Bulletin #14* (Zagreb 2018).

28 ‘Karamarko o kršenju zakona: Koji zakon? Pa to su branitelji,’ *Novi list* (29 May 2015), available at http://www.novilist.hr/Vijesti/Hrvatska/Karamarko-o-krsenju-zakona-Koji-zakon-Pa-to-su-branitelji (accessed 15 November 2018).

29 This quotation was located on a banner hung above the entrance to the Ministry of Veteran Affairs during the Tent Protest.

30 For a study on health and psychological problems suffered by Croatian war veterans, see V. Antičević, G. Kardum, and D. Britvić, ‘War Veterans’ Quality of Life: The Impact of Lifetime Traumatic Experiences, Psychological and Physical Health-Related Characteristics,’ *Društvena istrživanja*, 20, 4 (2011), 1101–18.

31 Medved, a regular participant at the tent protests, replaced Mijo Crnoja, the HDZ’s first choice for minister, who resigned after only six days at the job due to a scandal involving the misuse of veteran benefits.

32 For public perceptions of veterans based on results from focus groups, see I. Sokolić, *International Courts and Mass Atrocity: Narratives of War and Justice in Croatia* (Cham 2018), 141–64.

33 M. Špoljar, ‘Što rade i koliko javnog novca primaju 1,384 udruge branitelja,’ *Jutarnji list* (15 September 2018), 28. In 2017, the Ministry of Veteran Affairs alone provided veteran organizations with 17 million kuna (2.3 million Euros), which does not include the local sources of support. *Večernji list* (3 October 2017), 16.

34 *Večernji list* (6 October 2011), online version at www.vecernji.hr/vijesti/seks-nakon-rata-oruzjem-hrvatska-vodi-rat-promidzbom-clanak-333822 (accessed 16 March 2012).

35 For research into the political preferences of Croatia’s war veterans, see D. Bagić and K. Kardov, ‘Politička participacija i stranačke preferencije ratnih veterana u Hrvatskoj,’ *Politička misao*, 55, 3 (2018), 82–103.

36 B. Luci, ‘We Need to Insist on Elections Fought on Substance,’ *K2.0,* 9 June 2017, Available at: http://kosovotwopointzero.com/en/need-insist-elections-fought-substance/. (accessed 19 March 2019).

37 *Vijesti* (12 June 2017), online at http://www.vijesti.me/svijet/afp-o-izborima-na-kosovu-pobjedu-odnijela-koalicija-ratnika-941945 (accessed 19 March 2019).

38 A.L. Capussela, ‘Kosovo election: When the elites teamed up to see through their UÇK troubles,’ *LSE European Politics and Policy (EUROPP) Blog* (2017).

39 Author interview with Kosovo Specialist Chambers officials, The Hague, December 2016.

40 For histories of Kosovo and the Albanian national movement, see: T. Judah, *Kosovo: War and Revenge* (New Haven, CT 2002); M. Vickers, *The Albanians: A Modern History* (London 2011); N. Malcolm, *Kosovo: A Short History* (New York, NY 1999).

41 P. Gutaj and S. Al, ‘Statehood and the political dynamics of insurgency: UÇK and PKK in comparative perspective,’ *Journal of Balkan and Near Eastern Studies* 19, 2 (2017).

42 J. Pettifer, *Koncept pеür Realitetin e Ri. Dialog me Hashim Thaçin* [A Concept for a New Reality. A Dialogue with Hashim Thaçi]. (Prishtina 2001), 35.

43 I. Ströhle, ‘Veterans’ Politics and Policies towards the Veterans of the Kosovo Liberation Army,’ *Comparativ*, *Zeitschrift für Globalgeschichte und vergleichende Gesellschaftsforschung,* 20 (2010) Heft 5, S. 87–103.

44 A. Di Lellio and S. Schwandner-Sievers, ‘The Legendary Commander: the construction of an Albanian master narrative in postwar Kosovo,’ *Nations and Nationalism*, 12, 3 (2006), 513–29.

45 Schwander-Sievers shows how the UÇK is readily linked to older traditions of resistance among Albanians. S. Schwander-Sievers, ‘The bequest of Ilegalja: contested memories and moralities in contemporary Kosovo,’ *Nationalities Papers*, 41, 6 (2013), 953–70.

46 A. Bekaj, *The UÇK and the Kosovo War: From Intra-State Conflict to Independent Country* (Berlin 2010), 28.

47 V. Stojarová, ‘Albanian national armies – terrorists, guerrillas or national liberation movements?’, Institute for Comparative Political Research, Faculty of Social Studies Masaryk University, available at: http://ispo.fss.muni.cz/uploads/2download/Working_papers/ispo_wp_2006_11.pdf (accessed 19 March 2019).

48 Ibid.

49 Ströhle, ‘Veterans’ Politics and Policies towards the Veterans of the Kosovo Liberation Army’.

50 See https://www.transparency.org/country/KOS (accessed 19 March 2019).

51 D. Bilfesky, ‘Kosovo’s Thaci Aspires to Statesmanship, but Guerrilla Past Haunts Him,’ *New York Times* (12 July 2013), available at: https://www.nytimes.com/2013/07/13/world/europe/kosovos-thaci-aspires-to-statesmanship-but-guerrilla-past-haunts-him.html (accessed 19 March 2019). Also see N. Schmidle, ‘Bring Up the Bodies’, *The New Yorker* (6 May 2013), available at: https://www.newyorker.com/magazine/2013/05/06/bring-up-the-bodies (accessed 19 March 2019).

52 A. Qirezi, ‘New War Court Could Disrupt Kosovo’s Politics,’ *Balkan Insight* (2 November 2016), available at: https://www.balkaninsight.com/en/article/new-war-court-could-disrupt-kosovo-s-politics-11-01-2016 (accessed 19 March 2019).

53 Schmidle, ‘Bring Up the Bodies’.

54 S. Haxhiaj, ‘Kosovo’s Political Murders: Unpunished but Not Forgotten,’ *Balkan Insight,* 6 March 2018, available at http://www.balkaninsight.com/en/article/kosovo-s-political-murders-unpunished-but-not-forgotten-03-05-2018 (accessed 19 March 2019).

55 Schmidle, ‘Bring Up the Bodies’.

56 Qirezi, ‘New War Court.’

57 Ibid.

58 Schmidle, ‘Bring Up the Bodies’.

59 Ibid.

60 D. Morina, ‘Kosovo Court Releases Azem Syla From Custody,’ *Balkan Insight,* 12 March 2018, available at: http://www.balkaninsight.com/en/article/kosovo-court-releases-again-the-accused-politician-azem-syla-03-12-2018 (accessed 19 March 2019).

61 Ibid.

62 C. Sudetic, ‘The Bullies who Run Kosovo,’ *Politico* (21 July 2015), available at: https://www.politico.eu/article/kosovo-hashim-thaci-un-special-court-tribunal-organ-trafficking-KLA-serbia-milosevic-serbia-ramush/ (accessed 19 March 2019).

63 Quoted in Qirezi, ‘New War Court.’

64 Quoted in Bilefsky, ‘Kosovo’s Thaci Aspires to Statesmanship.’

65 Die Morina, ‘Kosovo ‘Sceptical’ About New Hague Court, Says PM,’ *Balkan Insight* (31 October 2018), available at: http://www.balkaninsight.com/en/article/specialist-chambers-seen-with-skepticism-kosovo-pm-says-10-31-2018 (accessed 19 March 2019).

66 See O. Stafasani, ‘Kosovo: Fake News in a Struggling Democracy’, *Eurozine* (29 September 2017), available at: http://www.eurozine.com/kosovo-fake-news-in-a-struggling-democracy/ (accessed 19 March 2019). The PDK and AAK, according to this account on an opposition website, have successfully co-opted many media figures and outlets, including previously independent journalists.

67 See CIA World Factbook, https://www.cia.gov/library/publications/resources/the-world-factbook/geos/kv.html (accessed 19 March 2019).

68 The* Guardian* (16 February 2018), available at: https://www.theguardian.com/world/2018/feb/16/kosovo-at-10-challenges-overshadow-independence-celebrations (accessed 19 March 2019).

69 F. Bytyci and K.Than, ‘Dramatic surge in Kosovars crossing illegally into EU’, *Reuters* (4 February 2015), available at: https://www.reuters.com/article/us-kosovo-eu-migrants/dramatic-surge-in-kosovars-crossing-illegally-into-eu-idUSKBN0L811120150204 (accessed 19 March 2019).

70 D. Morina, ‘Kosovo Parliament Approves Montenegro Border Deal’, *Balkan Insight* (21 March 2018), available at: http://www.balkaninsight.com/en/article/xx-kosovo-parliament-approves-montenegro-border-deal-03-21-2018 (accessed 19 March 2019).

71 D. Morina and M. Zivanovic, ‘Kosovo Imposes Customs Tariffs on Serbia, Bosnia,’ *Balkan Insight* (6 November 2018), available at: http://www.balkaninsight.com/en/article/kosovo-imposes-customs-tariffs-for-serbia-and-bosnia-and-herzegovina-11-06-2018 (accessed 19 March 2019).

72 RFE/RL, ‘Thousands Protest In Kosovo Over Possible Serbia Land Swap’ (29 September 2018), available at: https://www.rferl.org/a/protest-in-kosovo-over-possible-land-swap-with-serbia/29516478.html (accessed 19 March 2019).

73 Ströhle, ‘Veterans’ Politics and Policies towards the Veterans of the Kosovo Liberation Army,’ 94–6.

74 Ibid.

75 Quoted in Ströhle, ‘Veterans’ Politics and Policies towards the Veterans of the Kosovo Liberation Army,’ 97.

76 For an analysis of how armed groups transition into political parties, see C. Manning, ‘Armed opposition groups into political parties: comparing Bosnia, Kosovo, and Mozambique.’ *Studies in Comparative International Development*, 39, 1 (2004), 54–76.

77 It is worth mentioning that Yugoslav Partisan veterans of the Second World War were the beneficiaries of significant privileges during the communist period, which provides some context to the contemporary push for long-term veterans’ benefits.

78 *Novi list* (2 December 2015), 7.

